# FRET-Assisted Determination of CLN3 Membrane Topology

**DOI:** 10.1371/journal.pone.0102593

**Published:** 2014-07-22

**Authors:** Ewa Ratajczak, Anton Petcherski, Juliana Ramos-Moreno, Mika O. Ruonala

**Affiliations:** 1 Max-Planck Society Graduate School for Neurosciences, Göttingen, Germany; 2 Center for Membrane Proteomics, Goethe University of Frankfurt am Main, Frankfurt, Germany; University of Florida, United States of America

## Abstract

Juvenile neuronal ceroid lipofuscinosis (JNCL) is caused by mutations in the *CLN3* gene, which encodes for a putative lysosomal transmembrane protein with thus far undescribed structure and function. Here we investigate the membrane topology of human CLN3 protein with a combination of advanced molecular cloning, spectroscopy, and *in silico* computation. Using the transposomics cloning method we first created a library of human CLN3 cDNA clones either with a randomly inserted eGFP, a myc-tag, or both. The functionality of the clones was evaluated by assessing their ability to revert a previously reported lysosomal phenotype in immortalized cerebellar granular cells derived from *Cln3* ^Δex7/8^ mice (Cb*Cln3* ^Δex7/8^). The double-tagged clones were expressed in HeLa cells, and FRET was measured between the donor eGFP and an acceptor DyLight547 coupled to a monoclonal α-myc antibody to assess their relative membrane orientation. The data were used together with previously reported experimental data to compile a constrained membrane topology model for hCLN3 using TOPCONS consensus membrane prediction algorithm. Our model with six transmembrane domains and cytosolic N- and C-termini largely agrees with those previously suggested but differs in terms of the transmembrane domain positions as well as in the size of the luminal loops. This finding improves understanding the function of the native hCLN3 protein.

## Introduction

Neuronal ceroid lipofuscinosis (NCL, or Batten disease) refers to a group of lethal pediatric neurodegenerative diseases originating from mutations in one of the thus far identified 13 *CLN* genes (Ceroid Lipofuscinosis, Neuronal type; CLN1 to CLN14)[1]. The age of NCL onset varies between the different NCL forms but it is typically diagnosed in infancy or early childhood and terminates in premature death [2]. Physiological features common for most NCL forms include vision loss, motor and cognitive decline, as well as the progressive appearance of autofluorescent lysosomal storage bodies enriched in the subunit c of the mitochondrial ATPsynthase and in sphingolipid activator proteins [3]–[7]. The cells of the central nervous system (CNS) are primarily vulnerable in all NCL forms. Nevertheless, recently reported cardiac malfunctions in the murine model for juvenile form of NCL (JNCL) also suggest systemic responses outside the CNS [8]–[11].

JNCL is caused by mutations in the gene encoding for the CLN3 protein, the most common mutation in humans being a 1.02 kb deletion, which eliminates exons 7 and 8, and encodes for a truncated CLN3 protein [12]. CLN3 is a highly hydrophobic multi-membrane spanning protein with suggested lysosomal and endosomal localization. Data from patients and various JNCL models indirectly support a role for CLN3 in membrane trafficking, endocytosis and autophagy among others, as well as in the regulation of lysosomal pH and arginine transport [4], [13]–[16]. However, due to the lack of reliable tools the exact function and location of CLN3 remains unclear. Due to the extreme hydrophobicity and cross-species conservation, the generation of high fidelity CLN3 antibodies via conventional immunization has turned out to be difficult [17]. It is also unclear whether the epitopes used to generate peptide-specific antibodies are accessible in an intact cellular milieu [18]. To what extent the behavior of an ectopically expressed CLN3 fusion protein mimics that of endogenous CLN3 cannot be stated. However, earlier work has shown that ectopic expression of a full-length human CLN3 (hCLN3) protein is able to rescue a vacuolar phenotype in *S. cerevisiae Btn1*Δ knock-out model lacking the yeast orthologue of CLN3 [19], [20]. This demonstrates that ectopic expression of a modified CLN3 protein in a valid JNCL model can be used to test its functionality. In this study we exploited this observation and took advantage of a previously described lysosomal phenotype in cerebellar granule cells to test modified CLN3 proteins for their ability to revert this phenotype [13]. This genetically accurate mammalian cell culture model consists of immortalized cerebellar granule cells derived from CD1-*Cln3* ^Δex7/8^ mice homozygous for the most common human *Cln3* 1.02 kb deletion (Cb*Cln3* ^Δex7/8^)[14]

An accurate structure of CLN3 can only be retrieved using high-resolution methods, such as 3D cryo-EM or X-ray crystallography [18], [21]–[23]. These methods require substantial amounts of properly folded pure CLN3 protein in an adequate environment. Unfortunately, largely due to the absence of proper biochemical tools, attempts to isolate sufficient amounts of CLN3 from cells and tissues, or its production using *in vitro* methods have not been successful. At the moment, a model compiling a limited amount of experimental data and complemented by *in silico* predictions remains the consensus structural state of the art. This model describes CLN3 as a multi-membrane spanning protein with six transmembrane domains (TMDs), with both N- and C-termini facing the cytosol [17], [24], [25]).

In this work we experimentally investigated the membrane topology of tagged hCLN3 protein using Förster Resonance Energy Transfer microscopy (FRET). First, a library of 11 hCLN3 clones either with one internally inserted eGFP or myc-tag, or with a near C-terminal eGFP and one myc-tag towards the N-terminus, was created using the transposase cloning method. The functionality of the clones was evaluated by their degree of lysosomal targeting and their ability to rescue a specific lysosomal phenotype in the Cb*Cln3* ^Δex7/8^ cell line. The doubly-tagged hCLN3 clones were transiently transfected into HeLa cells, and labeled with DyLight-547 conjugated α-myc antibody. The FRET efficiency between eGFP donor and the DyLight547 acceptor was determined by Fluorescence Lifetime Imaging Microscopy (FLIM) and used to evaluate their relative membrane orientation. The FRET data were used in TOPCONS membrane prediction algorithm together with other available experimental data to serve as constraints for the computation of a novel hCLN3 membrane topology. The refined model for hCLN3 is in agreement with most of the available experimental data but differs from the previously suggested models in terms of the positions of the TMDs and the length of the cytosolic and luminal loops. Our model delivers intriguing new aspects to understanding the pathological mechanisms of JNCL.

## Results

### Generation and evaluation of internally tagged hCLN3 clones

**Figure 1:**
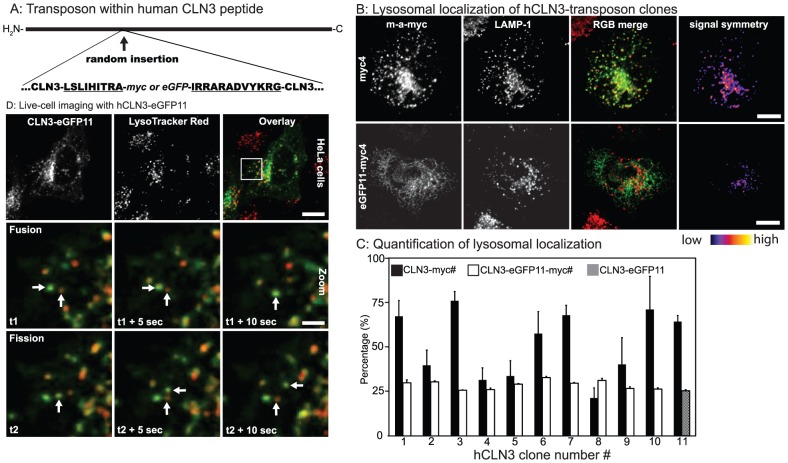
Generation of transposed hCLN3 clones. **A:** The transposon inserted within the human CLN3 coding sequence produces an hCLN3 protein with a randomly inserted eGFP flanked by 9- and 12- amino acid isolating peptides (underlined). See Table 1 for transposon insertion sites. eGFP in hCLN3-eGFP clones was later exchanged for a myc-tag while retaining the isolating peptide sequences. **B:** HeLa cells transfected with hCLN3-myc or –eGFP11-myc clones were counterstained for myc-tag and lysosomal membrane protein LAMP-1, and visualized with confocal microscopy, and the level of myc-tag colocalizing with LAMP-1 was analyzed using an intensity-independent colocalization algorithm (see Mat&Met). The image in the right-most column reflects the intensity symmetry between the myc-tag and LAMP-1. Scale bars 10 µm **C:** The level of expressed hCLN3 clones in the LAMP-1 positive lysosomes was quantified. Addition of an eGFP to the hCLN3-myc clones reduced the level of their lysosomal localization. Note that the last column for chimeras in C shows the colocalization degree of hCLN3-eGFP11 without myc-tag N = 2, n>3. **D:** HeLa cells expressing hCLN3-eGFP11 with the most C-terminal eGFP were counter-stained with LysoTracker Red vital dye (LTR), and live-imaged with confocal microscope at 0.2 Hz frequency. Shown is a crop from a 5 minutes image sequence illustrating the partial lysosomal localization of hCLN3-eGFP11 (top row). Regularly, hCLN3-eGFP11 positive vesicles were seen to fuse (arrows in middle row) or dissociate (arrows in bottom row) from LTR positive structures. Scale bars 10 and 2 µm, respectively.

A library of human *CLN3* cDNA clones with an enhanced Green Fluorescence Protein transposon and a kanamycin resistance cassette was created using the transposomics cloning technique using a pCMV5 plasmid with human CLN3 cDNA as the target vector. The resulting clones were examined in a three-step process to identify pCMV5-hCLN3 cDNA variants with a transposon within the hCLN3 sequence in the right orientation and reading frame (see Mat&Met)[26]. Following the removal of the kanamycin selection cassette from clones with successfully inserted transposon, the insertion sites were identified by sequencing (Table 1). The resulting plasmids encode for an hCLN3 with an internal eGFP flanked by 9 and 12 amino acid peptides [26], [27]. To generate tools for intramolecular FRET studies, we next exchanged the eGFP moiety in the first ten hCLN3-eGFP clones for a myc epitope (MEQKLISEED) while retaining the original insertion sites and the flanking peptide sequences. Subsequently, using fusion PCR method we created chimeric hCLN3-eGFP-myc clones by combining the myc-containing N-terminal half of the ten hCLN3-myc clones with the C-terminal half of the hCLN3-eGFP11 containing the eGFP. It is worth to mention that the nine nucleotide long repeats flanking each of the Tn5 insertions generated by staggered cuts of the transposase enzyme belong to the target DNA (Figure 1A).

**Table 1:** Original eGFP transposon insertion sites within the human CLN3 peptide.

clone ID	transposon after a.a.	Peptide sequence
1	36	HWK _transposon_HWKNAV
2	85	PHN_transposon_PHNSSS
3	101	AVL_transposon_AVLLAD
4	125	PYS_transposon_PYSPRV
5	150	VGT_transposon_VGTSLC
6	203	GLT_transposon_GLTQAG
7	238	AQD_transposon_AQDPGG
8	279	VFK_transposon_VFKGLL
9	345	RFT_transposon_RFTWAL
10	348	WAL_transposon_WALALL
11	406	HRE_transposon_HREFAM

The transposon encoding for eGFP was inserted into the pCMV5 expression vector containing the cDNA for the full-length human CLN3 amino acid sequence at indicated positions by transposase enzyme. The clone # is a running number for different clones as of the N-terminus, (left column), the middle column shows the number of the amino acid in the CLN3 peptide sequence after which the transposon was inserted, and the right column shows the corresponding hCLN3 amino acid sequence. eGFP in clones 1 to 10 was later replaced with a myc-tag and combined with hCLN3-eGFP11 to create chimeric clones with a near C-terminal eGFP and one myc-tag further towards the N-terminus. Refer to Figure 4A for visual location along the previously published model for hCLN3. Note that the last 3 amino acids before transposon insertion are duplicated behind the transposon insertion site (see Figure 1).

In order to investigate the functionality of the generated hCLN3 clones we first used the previously described lysosomal targeting as a criterion [17], [28], [29]. For this, HeLa cells transiently transfected with the hCLN3-myc and –eGFP11-myc clones were counterstained for myc-tag and the lysosomal marker protein LAMP-1. Using an intensity independent colocalization algorithm we found that the addition of a eGFP to the hCLN3-myc clones reduced the level of colocalization. Approximately a third of the chimeric clones colocalized with LAMP-1 positive structures (merge of signals, colocalization and signal symmetries are shown in Figure 1B, and the level of colocalization quantified in 1C. See Mat&Met for details)[30]. Interestingly, large proportion of the signal did not colocalize with lysosomes which is suggestive for a dynamic trafficking of the hCLN3. To investigate this HeLa cells expressing hCLN3-eGFP11 were co-stained with Lysotracker Red (LTR) vital dye and imaged live by confocal microscope. Time-lapse sequences showed dynamic CLN3-eGFP11 movement primarily in retrograde direction from the plasma membrane (a random snapshot frame illustrated in top row in Figure 1D; full movie sequence in online Movie S1). The level of colocalization with LTR varied constantly and regular fusion and budding events of hCLN3-eGFP11 with larger LTR positive structures took place (arrows in middle and bottom row in Figure 1B, respectively).

**Figure 2:**
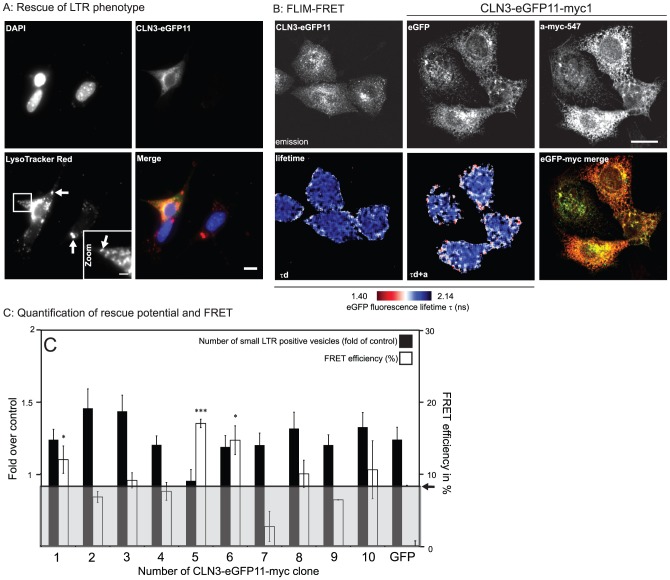
Evaluation of hCLN3 Clones and FRET analysis. **A:** *CbCln3* ^Δex7/8^ cells were grown under confluency for 7 days to produce autofluorescent storage material, transfected with hCLN3-eGFP11, or hCLN3-eGFP-myc clones and counterstained with LysoTracker Red (LTR). Shown are a cell expressing hCLN3-eGFP11 together with two non-transfected cells. Storage material accumulated in lysosomes is indicated by arrows in left panel of the bottom row. The number of small LTR positive vesicles in cells with or without exogenous hCLN3 expression (arrows in zoomed image) are quantified in C. Nuclei were stained with DAPI. Scale bars 10 µm for main panels, and 3 µm for the cropped image. **B:** HeLa cells expressing either hCLN3-eGFP11 or chimeric hCLN3-eGFP11-myc clones (shown is hCLN3-eGFP11-myc1) were fixed and stained with α-myc antibody directly conjugated with DyLight547. The fluorescence lifetime of the eGFP donor in the absence (τ_d_) or presence of the acceptor (τ_d+a_) was analyzed with a FLIM detector build on a confocal microscope (see Mat&Met), and the lifetime distribution in nanoseconds is shown in pseudo-color. The intensity images of the donor and acceptor were merged to demonstrate the expression levels and and myc- labeling efficiency. N = 2, n = 3–8. Scale bar 10 µm. **C:** Quantification of the rescue effect and FRET efficiency of hCLN3-eGFP11-myc clones (black and white columns, respectively). Small LTR positive vesicles in aged *Cln3* ^Δex7/8^ were quantified with the automated Cell Profiler image analysis platform, and expressed as a ratio against non-expressing cells. N = 3–5, n>45. Reduced fluorescence lifetime of the eGFP was converted to FRET efficiency and plotted against the myc-tag insertion site in hCLN3. The 8.4% cut-off for statistically significant FRET is greyed out and indicated (arrow).

When Cb*Cln3* ^Δex7/8^ cells are grown under confluency for a period of 7 days (aged), accumulation of autofluorescent lysosomal storage material, as well as altered number and morphology of LTR positive structures are observed (arrows in crop 2A)[13], [31]. We designed an experimental setup to test our transposed hCLN3-eGFP11-myc clones for functionality by measuring their potential to revert this LTR phenotype (Figure 2A). In comparison to non-transfected aged Cb*Cln3* ^Δex7/8^ cells expression of the hCLN3-eGFP11 without a myc-tag resulted in an increased number of small LTR positive vesicles (Figure 2A). Automated image analyses showed a 1.4 fold increase in the number of LTR positive vesicles in transfected cells and demonstrated the restoration of the LTR phenotype by correctly folded, functional hCLN3-eGFP11 (Figure 2A). The expression of hCLN3-eGFP-11 did not alter the number of small LTR vesicles in aged Cb*Cln3* ^+/+^ cerebellar granular cells (not shown). We next tested the rescue potential of chimeric hCLN3-eGFP11-myc clones. With the exception of hCLN3-eGFP11-myc5, all chimeric clones were able to increase the number of small LTR positive vesicles in aged Cb*Cln3* ^Δex7/8^ cells (Figure 2C). Together with the data on lysosomal targeting, this result indicates that, with one exception, the exogenously expressed, doubly-tagged hCLN3 fusion proteins are likely to fold in a proper manner,to retain at least partially the original function of CLN3, and to suit FRET-based structural studies.

### Investigating the membrane topology of hCLN3 chimeras by FRET-FLIM

The probability of Förster Resonance Energy Transfer (FRET) depends to the inverse sixth power on the donor-acceptor distance, a property which makes FRET a widely used method to investigate molecular proximity on a nanometer scale in intact cellular context [32]. One of the most accurate methods to determine FRET is to measure the characteristic fluorescence lifetime (tau; τ) of the donor fluorochrome using Fluorescence Lifetime Imaging Microscopy (FLIM) [33]. The distance dependency of FRET predicts that a separating membrane between donor and acceptor molecules would efficiently hinder the energy transfer. Based on this characteristic feature, we aimed to study the relative membrane orientation of the eGFP and myc-tag within hCLN3-eGFP11-myc clones. For this, we transiently transfected the chimeras into HeLa cells and stained the myc-tag with a monoclonal α-myc antibody directly labeled with DyLight547 as a FRET acceptor. The basic τ of the eGFP donor in cells expressing either the hCLN3-eGFP11 or the hCLN3-eGFP11-myc chimeras labeled with the α-myc-DyLight547 antibody was then determined. Although no detectable binding of the α-myc-DyLight547 antibody was observed in cells expressing the hCLN3-eGFP11 clone only, biexponential fit exposed the presence of two eGFP lifetime populations (τ_1_ =  2.28 ns±0.02, and τ_2_ = 2.14 ns±0.01). While the former τ_1_ represents the intrinsic lifetime of eGFP, the reduced τ_2_ is induced either by homo-FRET between neighboring eGFP molecules, or by nonspecific binding of the α-myc-DyLight547 antibody, and translates to a FRET efficiency of ∼4%. FLIM analysis on cells expressing hCLN3-eGFP11-myc chimeras and co-stained with α-myc-DyLight547 antibody exposed reduced mean τ_1_ ranging from 2.05 ns±0.04 to 1.71 ns±0.04 (Figure 2B, middle in bottom row). The reduction in τ_1_ varied for each chimeric hCLN3 clone and suggests a relation between τ_1,_ the insertion site of the myc-tag along the hCLN3 peptide and the membrane orientation of the myc-tag in respect to the eGFP donor (Figure 2C).

The Förster radius R_0_ describes an intramolecular donor-acceptor distance in which there is a 50% probability for FRET to occur and where the FRET efficiency is 50%. The statistically reliable range for FRET is defined by R_0_±50% R_0_, which corresponds to FRET efficiencies 8.4% and 91.2% [32]. The FRET efficiency for our chimeric hCLN3 clones was calculated from the reduction of fluorescence lifetime (τ) in donor-acceptor samples in comparison to τ of the donor-only controls. By applying the lower 8.4% threshold (greyed zone in Figure 2C) we identified chimeric clones yielding significant FRET. Based on the current consensus membrane topology models for CLN3 showing both the amino- and carboxyl-termini and amino acid 401 at the cytosolic side of the membrane, we assume an equally cytosolic location for the eGFP inserted after amino acid 406 [17]. Thus, myc-tags in chimeric clones with significant FRET are likely to share a cytosolic location with the eGFP. Clone hCLN3-eGFP11-myc1 (aa 37) produced a FRET efficiency of 12.0% ±1.9, indicating that both the eGFP and the myc-tag most likely reside at the cytosolic side of the membrane. The FRET efficiencies from hCLN3-eGFP11-myc5 (aa 151) and the hCLN3-eGFP11-myc6 clone (aa 204) clones of 17.0% ±0.6 and 14.7% ±2.0, respectively, suggest a cytosolic location for both of these myc-tag insertion sites. FRET from the remaining clones did not reach significance.

### Compiling FRET and previous data as a new model for hCLN3

**Figure 3:**
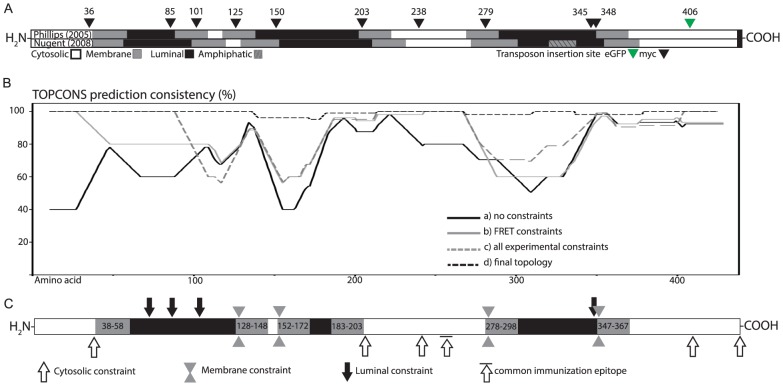
*In silico c*ompilation of FRET and other data as hCLN3 models using TOPCONS constrained membrane prediction algorithm. **A:** The transmembrane domains (grey), cytosolic (white) and luminal (black) loops from earlier hCLN3 models together with transposon insertion sites (black arrows for myc-tag, green for eGFP) are illustrated [25]. **B:** The reliability scores for CLN3 topology predictions indicating the consistency agreement between different prediction algorithms included in TOPCONS are shown as percentage. **C:** A schematic illustration of the new membrane topology obtained using constraints from FRET analysis, previously published experimental data, and the iterative approach. The cytosolic constraint with a line on top reflects the common epitope for two earlier used antibodies.

The two recent membrane topology models for CLN3 are based on multiple prediction algorithms constrained by available experimental data. Figure 3A illustrates our myc-tag and eGFP insertion sites in these models (Figure 3A; [25]). In 2009 Bernsel, *et al.* published TOPCONS prediction algorithm (from here on referred to as TC) which concludes the most probable membrane topology based on consensus between several individual algorithms [34]. TC performs a prediction for a given peptide sequence with SCAMPI-seq, SCAMPI-msa, PRODIV, PRO OCTOPUS and OCTOPUS prediction algorithms, and the provided reliability score for each amino acid reflects the agreement between different algorithms. Additionally, TC calculates the distance from the center of the membrane for each amino acid (Z-score), as well as the free energy value (ΔG) for membrane insertion [25], [34]–[36]. We decided to scrutinize our FRET data with a cycle of iterative TC predictions as described below. To set the starting point we first performed a constraint-free prediction for native hCLN3 peptide. The reliability score plot for the predicted structure with 10 transmembrane domains (TMDs; not shown) and equal location for both termini showed poor agreement between the used algorithms (line a in Figure 3B). Next, as suggested by FRET data, a prediction performed by constraining both tag loci of hCLN3-eGFP11-myc1 clone at the cytosolic side produced a topology structure with an increased reliability score and in line with existing experimental data (not shown) [17]. We continued by predicting a topology for the next positive FRET chimeric clone that was also able to rescue the lysosomal phenotype, hCLN3-GFP11-myc6. Here we set a cytosolic constraint for amino acids 407 (eGFP) and 204 (myc-tag), which in the previous prediction already had a cytosolic location. Except for the elongated cytosolic stretch between TM domains 4 and 5, the prediction showed no major difference in topology (not shown). Based on above data, we next set cytosolic constraints for the myc-tag and eGFP transposon insertion sites (aa 37), myc6 (aa 204) and eGFP11 (aa 407) for the native hCLN3 without the transposons. The resulting prediction showed a total of 9 TMDs with an improved reliability score when compared to constraint-free prediction (TMDs not shown; line b in Figure 3B). We finally tested how earlier experimental data would comply with the FRET-assisted model. We set additional cytosolic constraints for amino acids 250–258, 401, and the C-terminus of the active hCLN3 peptide, as shown by Kyttälä, Mao and Storch [17], [22], [23]. Additionally, we set luminal constraints for the amino acid residues 71 and 85 due to the reported N-glycosylation at these residues [23]. The resulting prediction showed 8 TMDs with two significant drops in reliability score around the second and third TMD and later along the hCLN3 peptide around amino acid 300 (TMDs not shown; line c in Figure 3B).

The close proximity of the myc-tag insertion in hCLN3-eGFP11-myc2 after amino acid 86 immediately after the glycosylation site at amino acid 85 suggest a possible correlation with luminal location and low FRET. By keeping the experimental constraints used for model above (c in Figure 3B) we tried to find the best possible location for the amino acid following the myc-tag of the remaining insertion sites individually. While a cytosolic constraint for amino acid 102 (myc3) failed to compute and a membrane constraint resulted in major decrease in reliability score, a luminal constraint produced a structure with a very high reliability score. The same pattern was observed using the myc-tag insertion site of clone hCLN3-eGFP11-myc4 (aa 126). A luminal constraint for amino acid 239 (myc7) failed to compute, but both membrane and cytosolic constraints resulted in high reliability scores. However, a membrane constraint introduced two novel TMDs and yielded a lower reliability score than a cytosolic constraint. Computations with a membrane constraint for the next insertion site (myc8, aa 280) produced the highest reliability score. Finally, the highest reliability scores for the last two insertion sites, myc9 and myc10, were obtained by setting a luminal constraint for amino acid 346 and a membrane constraint for amino acid 349.

We next proceeded to construct cumulative membrane topologies starting from either end of the CLN3 protein. When starting at the C-terminus we first set a luminal and a membrane constraint for amino acids 346 and 349 (myc9 and myc10, respectively). By keeping these constraints we evaluated all three constraints for amino acid 280 (myc8), and selected the membrane constraint producing the best score for the next prediction for analysis of amino acid 239 (myc7). Only the cytosolic constraint yielded a reliable prediction, which was kept for the prediction for amino acid 126 (myc4). Here, both cytosolic and membrane constraints produced a worse score than a luminal constraint. After adding a luminal constraint for amino acids 102 and 86 (myc3 and myc2, respectively) we finally obtained a prediction with high score for hCLN3 with six TMDs between amino acids 38–58, 128–148, 152–172, 183–203, 278–298, and 347–367 (Figure 3C). When the approach was repeated starting from N-terminus a similar topology was generated with TMDs between amino acids at 38–58, 124–144, 150–170, 183–203, 278–298, and 347–367 (Figure 3C). Predictions using a scrambled approach where the order of applied constraints was random equally resulted in topologies with six TMDs with minor variability in the starting amino acid of TMD 2 (not shown). Interestingly, a common feature for all of the predictions was a TMD between amino acids 152–172. Recalling the high FRET value but apparent impaired functionality of clone hCLN3-eGFP11-myc5, we speculated that the addition of a myc-tag after amino acid 150 might change the function but not necessarily the folding structure of hCLN3. Of all possible constraints for this insertion site we found that a membrane constraint produced the highest reliability score (d in Figure 3B).

We finally tested the possibility that sufficient number of constrains could force TC to produce a membrane topology with reasonably good score. For this we attempted to compute membrane topologies for hCLN3 using a collection 20 strings of constraints with 10 constraints each. Only one of the strings managed to compute successfully with a very low reliability score, which is indicative for the robustness of TC (not shown).

## Discussion

In this study we investigated the membrane topology of human CLN3 protein experimentally by measuring Förster Resonance Energy Transfer (FRET) between a FRET donor-acceptor pair inserted within the same CLN3 molecule. We assumed that location of both donor and acceptor on the same side of the membrane would result in measurable FRET, while location at opposing membrane sides would inhibit FRET. Chimeric CLN3 fusion proteins with an eGFP at amino acid 406 and one preceding myc-tag towards the N-terminus were generated using transposomics cloning strategy. The clones were first tested for functionality in HeLa cells using lysosomal targeting as criteria, and subsequently in Cb*Cln3* ^Δex7/8^ cells by determining their ability to complement impaired CLN3 function [13]. Finally, the chimeras were expressed in HeLa cells, and FRET efficiency was determined by measuring the fluorescence lifetime (FLIM) of the eGFP donor in the presence of a DyLight547-conjugated α-myc antibody. The FRET efficiency level of the different chimeras allowed us to draw conclusions about the relative membrane orientation of the donor-acceptor pair.

**Figure 4:**
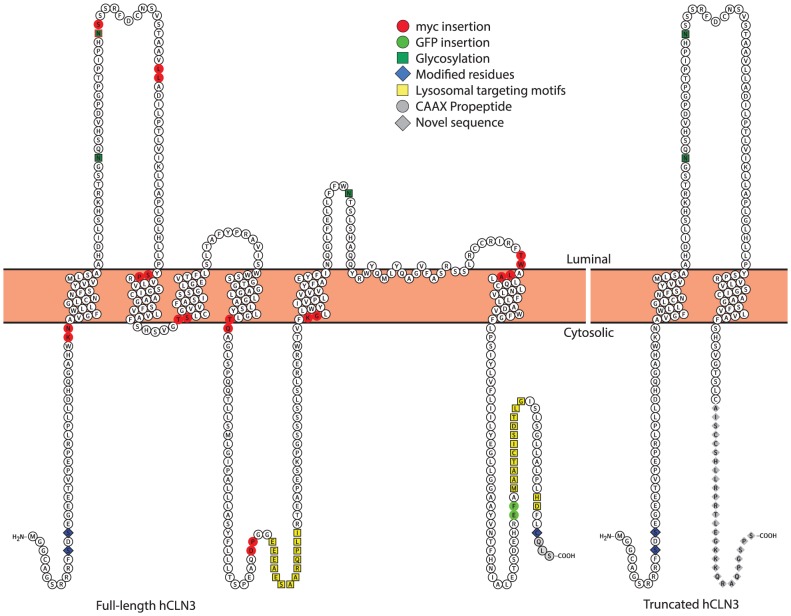
Illustration of the novel topology models of full-length and truncated forms of human CLN3. Myc-tag and eGFP insertion sites are illustrated on the new membrane topology model for the full-length human CLN3 (left) generated with Protter [39]. The corresponding topology of the most common truncated hCLN3 protein without insertion sites is shown on the right. The line indicates a common epitope used to generate antibodies used earlier [17], [21].

Statistically reliable FRET data were used to constrain the TC prediction together with other available experimental data, and the obtained topology was further refined with iterative cycles of TC predictions to generate a novel hCLN3 membrane topology with six transmembrane domains and cytosolic N- and C-termini (Figure 4). The generated model could accommodate almost all available experimental data with a very high degree of agreement score between all membrane predictions algorithms included in TC. Variations in length and starting point of TM domains two to four were accompanied by a slightly reduced score. The predicted topology has a C-terminal shift of the second TM domain, which in comparison to earlier models produces an extended first luminal loop [24], [25].

Our project was not hypothesis-driven, since we did not have any reasons to speculate whether there is a correlation between functionality of CLN3 and lysosomal targeting. Based on the validation data, however, it appears that lysosomal targeting alone does not guarantee functionality. Also, as we could not expect any chimeric clones to definitely produce FRET we did not have a ‘positive control', and thus the interpretation of our results follows the reasoning of logic. While some of the positive FRET data did agree with the existing topology models, such as that from chimeric clone myc1, our data could also demonstrate novelties in the hCLN3 structure. According to the earlier models chimeric clone myc5 with a luminal myc-tag inserted after amino acid 151 should not permit FRET; however, although insertion at this site apparently renders the clone non-functional it yielded the highest FRET readings. Also, the myc-tag in chimeric clone myc6 inserted after amino acid 203 should actually reside at the luminal side of the membrane; nevertheless, this clone was functional and produced significant FRET. Accumulating the positive FRET results from functional chimeric clones together with most of the other existing experimental data yielded a topology model in which these two insertion sites are close to the cytosolic sides of TMDs three and four, respectively [17], [22], [29]. Attempts to fit a model with a luminal N- and a cytosolic C-terminus returned an extremely poor reliability score. Low reliability scores also resulted from setting luminal constraints for amino acids 163–210 or 199. It is possible that the previously believed second TM represents an amphipathic helix partially embedded within the membrane at the luminal side.

Although numerous reasons could be envisioned our data offers no explanation for the very low FRET efficiency from clone hCLN3-eGFP11-myc7, which according to our model and those of others should indeed reside at the cytosolic side and thus be available to form FRET with eGFP. Possible explanations could be a nevertheless luminal location, an unfavourable relative dipole-dipole orientation of the antibody bound to the myc-tag with respect to eGFP, or a masking effect by protein-protein interactions. To test the possibility that additional transmembrane domains formed by two hydrophobic regions between amino acids 212–232 and 363–384 as suggested by a non-constrained TC prediction, by [25], but opposed by [17] would turn the myc7 at the luminal side and thus restrict FRET we attempted to compute a topology model with TC by constraining these regions as transmembrane domains. In any combination with our data above the addition of these regions as TM domains resulted in failure in computation (not shown). Only when most of the constrains we have obtained above were removed the TC managed to compute a topology with very low reliability score (not shown). Taken together, these points and the high reliability score for a cytosolic myc7 suggest other reasons as the source for low FRET, such as an unfavourable relative dipole-dipole orientation of the antibody bound to the myc-tag with respect to eGFP, or a steric hindrance effect by protein-protein interactions.

How closely our structural model truly reflects that of native hCLN3 can be evaluated when the structure of hCLN3 has been solved via high-resolution biophysical methods, e.g. X-ray crystallography. If our model would indeed prove to be correct, the C-terminal of the most common truncated form of hCLN3 arising from the 1.02 kb gene deletion would likely adopt a cytosolic instead of the thus-far suggested luminal orientation (right model in Figure 4). This may have interesting consequences in terms of understanding the suggested residual functionality the mutant CLN3, and its role in JNCL pathology [37]. Thus, the topology model presented in this study should be considered when designing, executing or evaluating future experimental work on the CLN3 protein, or in developing suitable tools for understanding details about the biological role of CLN3 and its mutations, especially in JNCL pathology.

## Materials and Methods

### Generation of transposed hCLN3 clones

A transposon encoding the enhanced green fluorescent protein (eGFP) and a kanamycin antibiotic selection cassette was amplified from template plasmid 24.6 (a kind gift of Dr. Douglas Sheridan [26]) with Accuzyme DNA polymerase (Bioline, Luckenwalde, Germany) with 20 PCR cycles using Tn5 primers

fw 5′-CTGTCTCTTATACACATCT-3′

and purified with Qiagen vacuum manifold (Qiagen, Hilden, Germany). 200 ng of pCMV5 cloning plasmid containing the human CLN3 gene between EcoRI and BamHI restriction sites (a kind gift of Dr. Ritva Tikkanen, University of Gieβen, Germany) was mixed with an equimolar ratio of the amplified transposon in transposase reaction buffer according to manufacturer's protocol (Epicentre, Madison, USA). 1 µl of transposase enzyme was added and the reaction mixture was incubated for 2 h at +37°C. The reaction was terminated by the addition of 2 µl of the stop solution. 1 µl reaction mixture was transformed into electro-competent OneShot *E*.Coli (Life technologies, Carlsbad, CA, USA) and plated on agar plates containing 100 µg/ml ampicillin and 50 µg/ml kanamycin (Roth Biochemicals, Karlsruhe, Germany). A PCR reaction was performed on double-resistant colonies using pCMV5-vector specific primers

fw 5′-CGTTGACGCAAATGGGCGG-3′, and

rv 5′-CCTCCACCCCATAATATTATAGAAGGACAC-3′

PCR from double-resistant colonies containing the transposon within hCLN3 coding sequence generated a ∼0.6 kb larger band than the ∼1.3 kb CLN3-pCMV5 from insertions outside the hCLN3 sequence. To verify the correct orientation of the inserted transposon, positive clones were analyzed via PCR using the pCMV5-fw primer described above and the eGFP-rv primer

5′-TTTACGTCGCCGTCCAG-3′

cDNAs of positive clones were amplified and inspected for a correct in-frame insertion by transient transfection into HeLa cells (see below). To remove the kanamycin selection cassette from cDNA clones producing visible eGFP signal the SrfI (Stratagene, La Jolla, CA, USA) digested plasmids were isolated by gel electrophoresis, religated using the T4 DNA Ligase (Thermo Fischer Scientific, Vilnius, Lithuania), and transformed into XL1-Blue *E.*Coli (Life technologies). Plasmid minipreps were prepared and the insertion site of the transposon was determined by sequencing using the GFP-rv primer described above. The transposon inserted within the hCLN3 peptide sequence consists of an eGFP flanked by 9 and 12 amino acid isolating peptides towards the N- and C-terminus, respectively. Additionally, the last three amino acids of the hCLN3 peptide sequence before transposon are replicated at the end of the transposon [26].

A myc-tag encoding the peptide sequence MEQKLISEEDL flanked by AscI restriction sites was produced by annealing a mixture with AscI-myc top and bottom oligos

5′-GGCGCGCCATGGAACAAAAACTAATAAGCGAAGAAGAC

CTAGGCGCGCC-3′

and used as a template in 25 PCR cycles using Phusion PCR polymerase (Finnzymes Oy, Espoo, Finland) with AscI-myc-fw and –rv primers

5′-GGCGCGCCATGGAACAAA-3′, and

5′-GGCGCGCCCTAGGTCTTC-3′

PCR products and the transposed hCLN3-eGFP clones were digested with AscI (SgsI, Fermentas, St. Leon-Rot, Germany), and purified with Millipore vacuum reaction clean-up plate (Millipore, Schwalbach, Germany). Inserts and vectors were religated using the T4 DNA Ligase. The orientations of the inserts in the XL-1 Blue transformed and ampicillin resistant colonies were investigated by PCR using pCMV5-fw and AscI-myc-rv and verified by sequencing. The 32 amino acid long polypeptide sequence of the complete myc transposon is

N-…CLN3_LSLIHIWRA**MEQKLISEEDL**IRRARADVYKRQ…CLN3-C

The chimeric CLN3-eGFP-myc clones were prepared using fusion PCR procedure. First, the CLN3 clones containing the myc insert were used as templates to amplify the first half of the CLN3 until amino acid 406 using the pCVM5-fw (above) and CLN3-mid-comp primers

fw 5′-GAGACAGCTCCCGGTGCTCATCACT-3′.

In parallel, the hCLN3-eGFP11 clone was used as a template in a PCR reaction with CLN3-mid-top

fw 5′-AGTGATGAGCACCGGGAGCTGTCTC-3′

and pCVM5-rv primers to amplify the second half of CLN3-eGFP. Next, PCR products were purified, mixed in equimolar ratios, and amplified by PCR using pCMV5 fw- and –rv primers. The produced PCR products encoding a myc- and eGFP-tagged hCLN3 were digested with EcoRI and BamHI (Thermo Fischer Scientific), and ligated into an empty pCVM5 cloning plasmid. Following ligation and transformation, plasmids were amplified and sequenced.

### Cell culture and transfections

HeLa cells were cultured in a humidified cell culture incubator (+37°C, 5% CO_2_) in DMEM medium (Life Technologies) containing 10% fetal calf serum (FCS, PAA Laboratories, Germany) and 1% Pen-Strep antibiotics (Life Technologies). Cultured cerebellar granular cells derived from CD1-*Cln3* ^Δex7/8^ mouse (a gift of Dr. Susan Cotman, Harvard Medical School, Boston, MA USA) were maintained in a humidified cell culture incubator (+33°C, 5% CO2) in granule neuron growth media (Dulbecco's Modified Eagle Medium, Life Technologies), 10% fetal bovine serum (Sigma-Aldrich, Munich, Germany), supplemented with 1% Pen-Strep antibiotics, 24 mM KCl and 200 ug/ml G418 Geneticin (Life Technologies).

For transfections MATra-A Reagent (IBA GmbH, Göttingen, Germany) was used. One day prior the transfection, HeLa or Cb*Cln3* ^Δex7/8^ cells were trypsinized and replated on #1 glass coverslips placed in 12- or 24-well plates at 2.0×10^5^ or 3.0×10^5^cells/well (for HeLa and Cb*Cln3* ^Δex7/8^ cells, respectively), and grown overnight. Next day cells were transfected with MATra method according to the manufacturer's protocol. In short, 0.1 µl of the MATra reagent and 5 µl of serum-free DMEM was added onto 100 ng of plasmid DNA/per cm^2^ of well area, and incubated for 20′ at RT. The total mixture was then added to the culture medium in the well plates. The plates were then placed on MATra magnet for 20′ at +37°C or +33°C (HeLa and Cb*Cln3* ^Δex7/8^ cells, respectively). 22 h post-transfection cells were either fixed (for confocal and FRET microscopy), or processed for live-cell imaging.

### Fluorescence microscopy

To prepare samples for confocal and FRET microscopy, HeLa cells were grown and transfected with appropriate plasmids using MATra-A Reagent as described above. 22 h post-transfection cells were washed four times with phosphate buffered saline (PBS, pH 7.4) and fixed with 4% paraformaldehyde (Sigma-Aldrich) in PBS for 30′. The samples were washed four times à 5′ with PBS, permeabilized for 5′ at RT with 0.2% Triton X-100 (Sigma-Aldrich), and quenched with 50 mM glycine for another 10′ (solutions in PBS). For antibody labeling the samples were blocked at RT for 30′ with 5% bovine serum albumin (BSA, Sigma-Aldrich) and 0.1% cold water fish gelatine (CWFG, Sigma-Aldrich) in PBS, and washed twice with incubation medium (0.5% BSA and 0.1% CWFG). Samples for FRET imaging were incubated with a 1∶500 in incubation medium diluted monoclonal α-myc antibody directly conjugated with DyLight547 according to the manufacturer's protocol (PIERCE, Offenbach, Germany). FRET samples were then washed multiple times with PBS, and mounted on glass slides with Mowiol-PBS (Mowiol-88, Sigma-Aldrich) without anti-fade reagent.

Samples for colocalization studies were incubated with polyclonal rabbit-α-LAMP-1 antibody (Sigma-Aldrich) diluted 1∶100, and/or monoclonal α-myc antibody diluted in incubation medium for 1 h at RT, washed with the same medium, and incubated with fluorescent secondary antibodies (goat-α-mouse Alexa-488, and goat-α-rabbit Alexa-568; Dianova, Hamburg, Germany) diluted in incubation medium for 1 h at RT. The cells were then washed four times with incubation medium, twice with PBS and mounted on glass slides with Mowiol-PBS without anti-fade reagent. The cells were visualized with a Leica SP2 AOBS confocal scanning laser microscope (Leica Microsystems, Heidelberg, Germany) using a 63×1.42NA oil immersion objective and appropriate laser excitation lines and emission windows with pinhole set at 1 Airy Unit and the photomultiplier amplification adjusted to avoid saturation.

For live-cell imaging HeLa cells were grown on glass-bottomed chamber slides (Nunc, Wiesbaden, Germany) to 30% confluency and transfected with MATra-A reagent as described above. Next day the cells were incubated for 30′ with LysoTracker Red DND-99 vital dye (LTR) diluted to a final concentration of 500 nM with cell culture medium, washed twice with imaging medium (DMEM without phenol red, 10% FCS, 1% PenStrep, 25 mM HEPES, (Life Technologies)), and transferred in the same medium to a custom-build, acclimatized live-imaging chamber warmed up to +37°C on a Leica SP2 AOBS confocal scanning laser microscope. The cells were visualized at 0.2 Hz using a 63×1.32 NA water immersion objective in line scan mode with appropriate excitation and emission settings.

### Rescue assay

Cb*Cln3* ^Δ*ex7/8*^ cells described earlier [13], were grown under confluency for 7 days to induce accumulation of storage material, trypsinized and counted. 3×10^4^ cells/well were seeded onto 13 mm #1 glass coverslips in a 24-well tissue culture plate, and incubated overnight. The following day, cells were transfected with 100-250 ng/cm^2^ of well area well of cDNA using MATra-A Reagent as described above. The next day the medium was supplemented with fresh medium containing LTR to a final concentration of 500 nM, and cells were placed back in the incubator for another 30′. Cells were washed four times with PBS, fixed as described above, followed by 10′ permeablization with digitonin (20 µg/ml in PBS), 10′ quenching with glycine (50 mg/ml), and 10′ incubation with a mixture of DAPI (1∶2000) and Alexa-647 conjugated Wheat Germ Agglutinin (both Life Technologies) in PBS. The samples were then washed four times for 5′ with PBS and mounted on glass slides with Mowiol as above. Samples were imaged in a single session with identical settings with either an Leica SP5 AOBS laser scanning confocal microscope (Leica), using a diode 405 laser for DAPI excitation, and Argon 488, HeNe 543 and 633 laser lines, for GFP, LTR and WGA-647 excitation, respectively, and a 63×1.42 NA oil immersion objective. Alternatively, samples were imaged with a Zeiss Axiovert 200 M epifluorescence microscope (Zeiss, Oberkochen, Germany) with appropriate excitation and emission filter sets for DAPI, GFP, LTR and Alexa-647, using a 16-bit MicroLine scientific CCD camera (Finger Lake Instruments, Rochester, NY, USA), and a 63×1.4 NA oil immersion objective. Using Cell Profiler image analysis software, small LTR positive vesicles (size range between 1-3 µm) were identified and counted for each cell in a visual field, and correlated with the presence or absence of eGFP expression [38]. Both imaging systems gave comparable results, and the number of small LTR positive vesicles in transfected cells was expressed as a ratio against the number of small LTR positive vesicles counted in non-transfected cells. Data shown are the average of at least three repeated analyses (N = 3-5for each clone) with at least 15 transfected cells per data set (n = 45-75). Statistical analysis was performed with ANOVA and error bars represent the standard error of the mean (SEM). The Cell Profiler software and the used analysis pipelines can be obtained from http://www.cellprofiler.org.

### Mathematical colocalization analysis

The colocalization of signal in two different confocal channels was calculated with an image asymmetry correlation algorithm allowing unbiased analysis [30]. In short, the images for each channel were imported into MatLab as 8-bit images, normalized to maximum and filtered with 3×3 pixel Wiener 2D Kernel and 3×3 pixel averaging. Zero blanking was applied and the threshold was set at 10 counts for the colocalization image. The fraction of pixels colocalizing with LAMP-1 was plotted as a function of myc-tag insertion site, Student's t-test was used to evaluate the significance, and error bars represent SEM.

### Fluorescence lifetime imaging and FRET analysis

To determine the fluorescence lifetime (tau, τ) of the donor fluorochrome (eGFP) samples were excited with a Coherent MIRA (Coherent, Göttingen, Germany) pulse intensified femtosecond two-photon laser set at 900 nm. The emission was collected using a 505/30 BP filter and a 63×1.42 NA oil immersion objective for 10′ with a Time-correlated Single Photon Counting unit (TCSPC, Becker&Hickl GmbH, Berlin, Germany) coupled to a Leica DM IRE2 AOBS inverted confocal microscope (Leica). The τ distributions in each image were analyzed with TCSPC analysis software v.2.4 (Becker&Hickl) by binning the raw signal with a factor of 5, pixels with less than 25 counts were filtered out as background fluorescence, and the lifetime decay fit of the donor was calculated using a biexponential fit. The lifetime data (τ1 and τ2) were imported to Origin Pro 7.0 statistical analysis software (OriginLab Corp., Northampton, MA, USA) and a single Gaussian fit was used to determine the peak of both lifetime populations. FRET efficiency in donor-acceptor samples was calculated according to the formula: %FRET = (1-(τ_donor_+_acceptor_/τ_donor_))*100.

using the lower lifetime population of the donor as a reference. The significance of variation between the donor and other samples was calculated by applying two-tailed Student's T-test for heteroscedastic data sets (unequal variances). Variation within equal samples was calculated via Student's T-test; paired or for heteroscedastic data sets (unequal data sets). Error bars represent SEM.

### TOPCONS membrane domain prediction algorithm

The web-based consensus membrane topology prediction algorithm TOPCONS was used according to on-line instructions, and can be accessed at http://topcons.cbr.su.se/ [34]. From each attempted prediction an image describing the transmembrane domains and reliability score was plotted, and a data file with computed parameters saved for further inspection.

## Supporting Information

Movie S1 **The movie shows the movement of hCLN3-eGFP11 expressed in HeLa cells (left panel) together with LysoTracker Red (LTR; middle panel) at 0.2 Hz frequency.** The merge is shown in the right panel. (online)
(AVI)
